# Robot‐assisted PSMA‐radioguided surgery for local recurrence in the prostatic bed

**DOI:** 10.1111/bju.16770

**Published:** 2025-05-19

**Authors:** Fabian Falkenbach, Giovanni Mazzucato, Marie‐Lena Schmalhofer, Zhe Tian, Pierre I. Karakiewicz, Markus Graefen, Lars Budäus, Philipp Mandel, Fijs W.B. van Leeuwen, Matthias N. van Oosterom, Anne‐Claire Berrens, Henk G. van der Poel, Farzad Shenas, Daniel Koehler, Sophie Knipper, Tobias Maurer

**Affiliations:** ^1^ Martini‐Klinik Prostate Cancer Center University Medical Center Hamburg‐Eppendorf Hamburg Germany; ^2^ Department of Diagnostic and Interventional Radiology and Nuclear Medicine University Medical Center Hamburg‐Eppendorf Hamburg Germany; ^3^ Department of Urology University Medical Center Hamburg‐Eppendorf Hamburg Germany; ^4^ Department of Urology Vivantes Klinikum am Urban Berlin Germany; ^5^ Cancer Prognostics and Health Outcomes Unit, Division of Urology University of Montréal Health Center Montréal Québec Canada; ^6^ Department of Urology, Azienda Ospedaliera Universitaria Integrata di Verona University of Verona Verona Italy; ^7^ Interventional Molecular Imaging Laboratory Leiden University Medical Center Leiden The Netherlands; ^8^ Department of Urology Netherlands Cancer Institute‐Antoni van Leeuwenhoek Hospital Amsterdam The Netherlands; ^9^ Department of Urology Amsterdam University Medical Centers Amsterdam The Netherlands

**Keywords:** Biochemical recurrence, prostate‐specific membrane antigen, seminal vesicles, prostate cancer, prostate‐specific antigen, radioguided surgery

**Abbreviations**
BCR(FS): biochemical recurrence (‐free survival)
CT: computed tomography
IQR: interquartile range
PCa: prostate cancer
PFS: progression‐free survival
PSMA: prostate‐specific membrane antigen
RGS: radioguided surgery
RP: radical prostatectomy
RT: radiation therapy
TFS: treatment‐free survival

Prostate‐specific membrane antigen (PSMA)‐imaging revealed evidence of locally confined relapses in one‐quarter of patients with primary biochemical recurrence (BCR) after radical prostatectomy (RP) [1]. PSMA‐targeted radioguided surgery (RGS) can facilitate precise removal of these lesions [2] as an alternative to salvage radiation therapy (RT). While open PSMA‐RGS has demonstrated its potential for nodal disease [3] and local recurrences [2], the robotic adaptation of the latter has not yet been examined.

For analysis, we included all consecutive non‐metastatic patients treated with robot‐assisted PSMA‐RGS for local prostate cancer (PCa) recurrence within the prostatic bed/fossa, prostatic vascular pedicle, remnants of the seminal vesicles, or the surrounding soft‐tissue, identified by PSMA‐imaging after BCR post RP. Following prior research [2, 3], we recorded the biochemical response, BCR‐free survival (BCRFS, PSA <0.2 ng/mL), and treatment‐free survival (TFS). The Kaplan–Meier method estimated survival rates. All tests were two‐sided, with the significance level set at *P* < 0.05.

The day prior to surgery, [^99m^Tc]Tc‐PSMA‐I&S was administered intravenously, and tracer uptake was verified by single‐photon emission CT/CT prior to surgery. Patients were placed in a 30° Trendelenburg position, a urinary catheter was inserted, and trocars were positioned in the same manner as for robot‐assisted RP (Fig. 1). After accessing the abdominal cavity, adhesiolysis was performed if necessary. The bladder was irrigated with a saline solution to minimise ^99m^Tc contamination. The bladder was then mobilised ventrally, and the rectovesical space developed. Using a miniaturised gamma probe (Drop‐in probe CXS‐OP‐DP; Crystal Photonics, Berlin, Germany; or SENSEI probe by Lightpoint, a Telix Pharmaceuticals Company, London, UK) grasped by the ProGrasp™ forceps (Intuitive Surgical, Sunnyvale, CA, USA) the area of interest was systematically scanned in a meander‐like pattern to precisely localise and define the extent of the cancerous lesion. Next to the lesion, the peritoneal surface overlying the dorsal bladder was incised. To reduce the risk of ureteric injury, an incision was made along the ductus deferens and medial to the ureter. Further development of this space directly leads to the peritoneal fold anterior to the rectum and posterior to the bladder. The cancerous lesion was resected *en bloc*, retrieved within a specimen retrieval bag through the camera port, and evaluated for cancer by *ex vivo* measurements. After dissection, the gamma probe was reintroduced, and the resection bed was again systematically scanned to verify the complete removal of the cancerous tissue. If radioactivity remained (counts/s higher than at least twice the background measurements), further resection was prompted which happened in two cases.

**Fig. 1:**
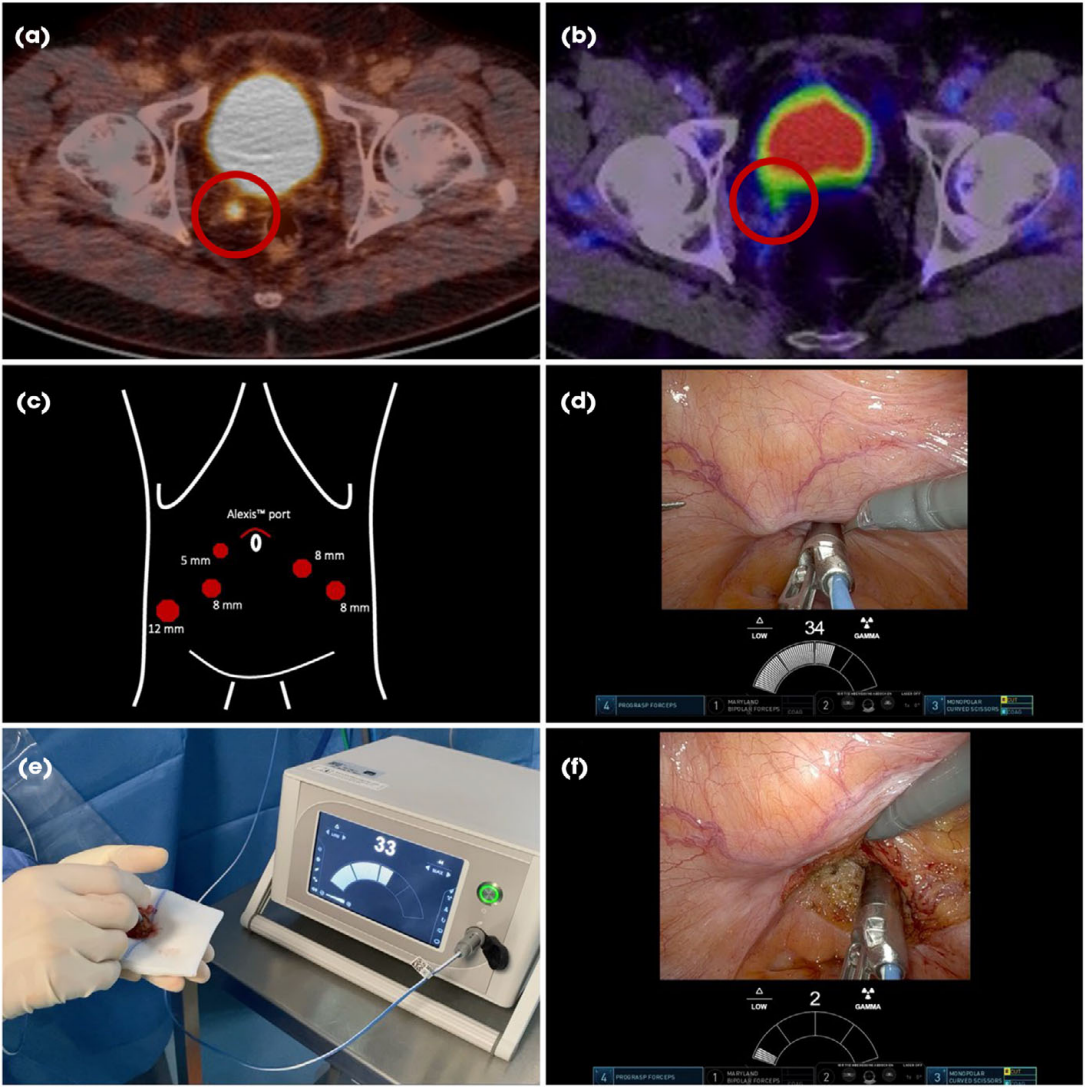
Workflow of robot‐assisted, RGS for local recurrences: (**a**) PSMA PET/CT fusion imaging identifies eligibility, (**b**) preoperative [^99m^Tc]Tc‐PSMA‐I&S single‐photon emission CT/CT fusion imaging to cross‐validate tracer uptake, (**c**) trocar placement for surgery, (**d**) *in vivo* assessment of the localisation and extension of the local recurrence using a drop‐in gamma‐probe, (**e**) confirming the removal of cancerous tissue by *ex vivo* measurements using the same gamma‐probe, (**f**) verifying no residual cancer tissue by systematically scanning the resection bed. See accompanying Video S1.

Of 54 robot‐assisted PSMA‐RGS procedures between 2021 and 2024, we identified 17 (31.5%) consecutive patients who underwent PSMA‐RGS for local recurrences. At the initial RP, most patients presented with advanced stage (≥pT3: 10 of 17 [59%]) and adverse Gleason Grade Group ≥3 (nine of 17 [53%]). The initial RP was performed using an open approach in two patients. At PSMA‐RGS, the median (interquartile range [IQR]) age and PSA level were 63 (61–67) years and 0.54 (0.42–0.82) ng/mL, respectively. Most patients presented with high‐risk BCR according to European Association of Urology BCR risk stratification (12 of 17 [71%]). More than one‐third of the patients (six of 17) underwent PSMA‐RGS after initial RP and RT. Of these, three of 17 (18%) and three of 17 (18%) patients received local adjuvant and salvage RT, respectively. Follow‐up information was available for all patients at a median (IQR) of 12 (6–24) months. Pathological examinations confirmed the removal of cancerous tissue in 15 of 17 (88%) patients. In the first patient without removed cancer, subsequent PSMA‐imaging revealed initial false‐positive findings in the prostatic fossa and an additional true positive sclerotic rib lesion. In the second patient without cancer removal, subsequent PSMA‐imaging revealed progressive local recurrence, which we failed to remove. PSA levels decreased in 14 of 17 (82%) patients to a median (IQR) of 0.13 (0.02–0.30) ng/mL. Biochemical responses of <0.1, 0.1–<0.2, and ≥0.2 ng/mL were observed in eight of 17 (47%), four of 17 (24%), and five of 17 (29%) patients, respectively. The 1‐year BCRFS and TFS was 34% (95% CI 13–86%) and 93% (95% CI 80–100%), respectively. In patients with a PSA level <0.1 ng/mL after PSMA‐RGS, no BCR or further treatment was recorded during follow‐up. The median (IQR) operative time was 80 (72–100) min. The median (IQR) estimated blood loss was 20 (10–50) mL. One severe complication occurred (thermal ureter injury requiring ureter reimplantation).

Therapeutic options for local recurrence of PCa after RP are limited, especially if prior RT has been administered. Robot‐assisted PSMA‐RGS offers a promising new addition to the armamentarium for robotic surgeons. It supports salvage resection of local diseases with a reasonable safety profile and promising biochemical responses.

First, the PSA level declined in 14 of 17 (82%) patients. A complete biochemical response below a PSA level of <0.2 ng/mL was observed in 12 of 17 (71%) patients. In comparison to our initial series utilising the open approach, immediate oncological results were comparable (complete biochemical response rate of 78%) [2]. The 1‐year BCRFS and TFS rates were 62% (vs 34%) and 88% (vs 93%), respectively. Giesen et al. [4] recently reported results of open vs robot‐assisted salvage vesiculectomy for local PCa recurrences in the remnants of seminal vesicles with comparable results. In the subgroup of patients with primary RP, a PSA level decline of <0.1 ng/mL was reported in 35% (vs 47%, in our study) and cancerous tissue was removed in 89% (vs 88%, in our study) of patients. Still, two patients without removed cancer and an overall 1‐year BCRFS of 34% in the present analysis are concerning and demand further investigation.

Second, patients with a PSA level of <0.1 ng/mL after surgery (8 of 17 [47%]) did not experience any recurrence or further PCa treatments during follow‐up. This underlines the assumption that metastases may originate primarily from local recurrences in a certain subset of patients. Metastatic progression may be stopped by salvage surgery, when no occult metastases exist, that may present later in imaging but are detectable by a residual PSA level ≥0.1 ng/mL after PSMA‐RGS. Similarly, Knipper et al. [3] recently reported an excellent 2‐year TFS rate of 81% in patients with a biochemical response with a PSA level of <0.1 ng/mL after PSMA‐RGS for any oligorecurrent PCa (almost exclusively pelvic nodal metastases) from a large multicentre cohort. This heterogeneity of cancer control outcomes after salvage surgery illustrates the need for improved patient selection, which is currently being actively investigated (e.g., the BioPoP trial on predictive biomarkers, ClinicalTrials.gov identifier: NCT04324983).

Third, one‐third of the patients had undergone prior RT. Literature on local therapies for patients with local recurrence after RP + RT is scarce [5]. This question was explicitly discussed in the *Advanced Prostate Cancer Consensus Conference* 2024 meeting, and no consensus has been reached concerning the best treatment modality [6]. This illustrates both the clinical necessity and the uncertainty of this topic. Although prior RT after RP is classically considered a contraindication for RT, several small trials have investigated re‐irradiation after RP + RT and reported 1‐year progression‐free survival (PFS) around 80% [7]. Re‐irradiation may be considered the only true alternative to PSMA‐RGS in these patients. For example, Archer et al. [8] conducted a retrospective multicentre analysis of 117 patients treated with re‐irradiation for local recurrence after RP + RT. The 1‐year PFS was 74% (vs 1‐year TFS rate of 92% in the present study).

Our analysis was limited by its retrospective design, small sample size, and the absence of a control group. Moreover, the follow‐up of the present study was too short to draw conclusions on the long‐term efficacy.

## Funding

The PSMA‐RGS procedure was performed as part of routine clinical care. The SENSEI gamma probes were provided by Lightpoint, a Telix Pharmaceuticals company.

## Disclosure of Interests

The authors declare that they have no conflicts of interest relevant to the content of this manuscript. All authors directly participated in the planning, execution, or analysis of the study. This research received no specific grants from any funding agency in the public, commercial, or not‐for‐profit sector. The authors have no relevant financial interests to disclose.

## Ethics Approval

This study was conducted in accordance with the ethical standards of the institutional and national research committee and with the 1964 Helsinki Declaration and its later amendments. This retrospective analysis was approved by the institutional review board of Hamburg, Germany (2019‐PS‐09; PV7316).

## Informed Consent

All patients were informed about the experimental nature of salvage surgery and the use of [^99m^Tc]Tc‐PSMA‐I&S for PSMA‐RGS, consented to the procedure, and allowed data use for research. All data were prospectively stored in an institutional database (FileMaker, Claris International Inc., Cupertino, CA, USA).

## Supporting information


**Video S1.** Robot‐assisted PSMA‐radioguided surgery for local recurrent prostate cancer after radical prostatectomy.

## Data Availability

Data are available for bona fide researchers who request it from the authors upon reasonable request.
